# Editorial: The emergence of animal welfare science and policy in Africa, Asia and Latin America

**DOI:** 10.3389/fvets.2023.1171229

**Published:** 2023-03-28

**Authors:** Jeremy N. Marchant, Rebecca E. Doyle, Maria José Hötzel, Oluwaseun S. Iyasere, Michelle Sinclair

**Affiliations:** ^1^United States Department of Agriculture - Agricultural Research Service, Livestock Behavior Research Unit, West Lafayette, IN, United States; ^2^Animal and Human Health Program, International Livestock Research Institute, Addis Ababa, Ethiopia; ^3^Royal (Dick) School of Veterinary Studies, University of Edinburgh, Edinburgh, United Kingdom; ^4^Laboratório de Etologia Aplicada e Bem-Estar Animal, Universidade Federal de Santa Catarina, Florianópolis, Brazil; ^5^Department of Animal Physiology, Federal University of Agriculture, Abeokuta, Nigeria; ^6^Albrecht Daniel Thaer-Institut für Agrar- und Gartenbauwissenschaften Tierhaltungssysteme und Ethologie, Humboldt University, Berlin, Germany; ^7^School of Veterinary Science, University of Queensland, Brisbane, QLD, Australia; ^8^Animal Law and Policy Program, Harvard Law School, Cambridge, MA, United States

**Keywords:** animal behavior, animal welfare, animal welfare policy, developing countries, extensive systems, local breeds

As part of efforts to raise the profile of animal welfare science within Africa, Asia and Latin America, this Research Topic was generously supported with full article processing charge remission by Frontiers Media SA, to enable scientists to publish their work in a high-quality Open Access journal. For many countries within these regions, animal welfare science is still nascent and this Research Topic highlights some of the animal welfare issues within these regions and the local scientific research being directed to find solutions. The result is a diverse collection of papers covering farm, laboratory and zoo animals.

Animal welfare science as a discipline, has a relatively modern history. Although good treatment of animals is an important tenet of some religions and civilizations dating back a few millennia, for example the concept of Ahimsa in Jainism, Buddhism, Hinduism, and Sikhism (1), the formation of policy and enactment of legislation has almost exclusively been a 20th Century and onwards phenomenon. Although the first known animal protection legislations were passed in Ireland and the Massachusetts Colony in 1635 and 1641, respectively (2), and anti-cruelty legislation for cattle and other animals passed in the U.K. in 1822 and 1876, the catalyst for more widespread welfare-focused legislation and for the emergence of animal welfare science was Ruth Harrison's book Animal Machines (3) and the subsequent Brambell Report established by the UK Government (4).

Within the Brambell Report was the embryonic text of what evolved into the Five Freedoms, and also Appendix III (5) which detailed the scientific assessment of pain and distress in the principal farm animal species. From these acorns, animal welfare as a scientific specialty grew, though not without growing pains and indeed still some suspicion from some veterinarians and animal scientists in particular. With the entry of the UK into the European Union in 1973, animal welfare became an EU-level issue (6), with formation and expansion of funding for animal welfare science, and formation of advisory bodies, ultimately the European Food Safety Authority, to collate, interpret and report on the science of animal welfare in order inform policy and legislation. With the appointment of Prof. Donald Broom to the world's first Chair in Animal Welfare in 1986 at the University of Cambridge, animal welfare science began its introduction into veterinary teaching, spreading across Europe and gradually further afield across the rest of the world. The World Organization for Animal Health began incorporating animal welfare into the Terrestrial Animal Health Code (7) in 2004, meaning 182 member countries across the world have approved the concept of animal welfare and the development and implementation of animal welfare standards. It is clear that animal welfare, and laws to protect animals, are important across the world (8).

However, it has been suggested that the historical spread of “Western” farming methods represented animal colonialism, defined as “a dual phenomenon, consisting, on the one hand, in using animals to colonize lands, native animals, and people and, on the other hand, in imposing foreign legal norms and practices of human-animal relations upon communities and their environments” (9). Therefore, as animal welfare science expands globally, we must be cautious that it retains its relevance to cultural issues, and that a “euro-centric” focus of animal welfare defined by its evolutionary origin is not imposed upon other cultures, in a form of neocolonialism (10). The answers to animal welfare issues within Africa, Asia and Latin America lie within these areas. Although we continue to see the spread of intensive farming systems and other animal uses into these regions (11), the animal welfare issues may be familiar ones, but may also be different. It is imperative that internal and external stakeholders invest in animal welfare science inside these geographic areas, both in terms of people—animal welfare scientists, lecturers, auditors, etc.—and infrastructure, and that local and national animal welfare issues are primarily addressed by local and national expertise.

A cursory Web of Science Core Collection search of the term “animal welfare” yields just under 25,000 papers. Of these, around 1,800 have authors based Latin America, 1,400 have authors based in Asia (excluding Japan) and 500 have authors based in Africa, illustrating the relative strengths of animal welfare science in the regions. This may also be reflected by the degree of collaboration with coauthors from outside the region. Although collaboration with scientists external to the region could have benefits in terms of English language publishing (Gallo et al.) and reducing conscious and unconscious biases in the publishing process, reduced collaboration can also indicate that animal welfare science is more established and that there is less need to collaborate. About 65% of papers from Latin America and Asia have within-region coauthors only but this drops to 40% for papers from Africa. For papers specifically addressing animal welfare within these regions, under 10% of papers concerning Latin America and Asia have no authors from those regions, but this increases to nearly 25% for animal welfare within Africa—i.e., a quarter have no local expertise input.

There is ongoing intensification of animal agriculture within all regions of the Research Topic (12), and the introduction of highly-selected breeds. This may result in a potential loss of indigenous breeds which are not only the mainstay of small-scale production—providing income and nutrition—but are also a valuable genetic resource (13). A better understanding of their behavior and welfare can impact survivability and efficiency of production, with corresponding human benefits, thereby safeguarding their preservation. A pair of papers on Nigerian indigenous chickens investigated differences in maternal care of hens and fear responses of chicks of two ecotypes (Oyeniran et al.) and the hens' responses to visual or physical separation from their chicks (Iyasere et al.). Both of these papers help to identify behavioral traits that might improve survival within the extensive systems in which the chickens are kept, with frequent exposure to predation. Also, increasingly important within Africa is aquaculture, with the two dominant species being tilapia and African catfish. Ojelade et al. investigated the impacts of providing environmental enrichment to catfish under laboratory conditions, and found advantages in growth rates and reduced aggression, warranting further research to determine potential application to commercial fisheries.

Animal welfare can only be improved with knowledge. This includes knowledge of the current status of the animal's welfare, knowledge of people's current perceptions of, and attitudes toward animal welfare (8), and knowledge about barriers that may be preventing adoption of ideas or mechanisms that may improve welfare, specific to the culture in which improvement is trying to be enacted (14). Assessment of current welfare is a good starting point from which to enact change. Romero et al. used a previously standardized and validated protocol with animal-based measures of behavior and health to assess welfare of horses and mules in Colombia by direct observation. Racciatti et al. developed a welfare assessment protocol including animal-, resource- and management-based measures that could be used across multiple zoo animal species, including mammals, birds and reptiles, again by direct observation. Resasco and Diaz surveyed laboratory mice breeding facilities in Argentina, using animal-, resource- and management-based measures to provide the first knowledge about welfare within such facilities. These assessments yield important information that can then be used to highlight areas of concern, develop training to address identified issues and inform future direction.

Lemma et al. explored animal welfare perceptions in rural households in Ethiopia using a Community Conversations methodology, using facilitated group discussions to identify community strengths and constraints, values and practices and explore strategies to address livestock management challenges. A survey of Ethiopian livestock-owning households is reported in Alemayehu et al., using a survey tool designed to measure participants' Knowledge, Attitude and Practice (KAP) of animal welfare. Community Conversations raise awareness and can serve as an effective way to channel community feedback into welfare improvement programs. The KAP methodology can help identify areas requiring targeted training. A survey of egg producers from 6 Asian countries (de Luna et al.) explored the benefits and challenges to adopting cage-free systems, showing that there is a widespread perception that caged systems have cost and ease of management advantages, but that cage-free systems are perceived as higher welfare. Nearly three-quarters of producers said more support is needed to establish cage-free farms, with technical advice, training and resources needed.

Another area of animal welfare that has received increasing scrutiny over the last few years is that of animal tourism. In some developing economies, these activities can be seen as important drivers of income into the country in general, as well as obviously directly impacting individual livelihoods. The COVID-19 pandemic brought travel and tourism to a halt, impacting human and animal welfare. Supanta et al. examined the impacts of COVID-19 on elephant camp management in Thailand, and the reduction in income lead to unemployment of carers, which itself could impact elephant welfare, and increased time spent chained and decreased nutrition.

Finally, the trends in farm animal welfare publications in Latin America were examined by Gallo et al.. Over the last 30 years, nearly 700 papers were identified on farm animal welfare produced by researchers in Latin American countries. However, 95% were published in the last 15 years, showing a rapid increase during this time, both in research and in training. Nearly 70% were produced by Brazilian and Mexican researchers and over 40% were on cattle, illustrating the importance of these countries cattle industries.

Overall, the quantity and quality of research being carried out in Africa, Asia and Latin America is increasing. The notion that animal welfare is important to more developed countries alone is false (8), and we must regard animal welfare as a key factor within sustainability and development frameworks (15), as we seek to improve the lives of all human and non-human inhabitants of our planet.

## Author contributions

All authors listed have made a substantial, direct, and intellectual contribution to the work and approved it for publication.

## Author disclaimer

The findings and conclusions in this publication are those of the authors and should not be construed to represent any official USDA or US Government determination or policy. The mention of any trade name, proprietary product, or specific equipment does not constitute a guarantee or warranty by USDA-ARS and does not imply its approval to the exclusion of other products that may also be suitable. The USDA-ARS is an equal opportunity and affirmative action employer. Lastly, all agency services are available without discrimination.
